# Evolution in fecal bacterial/viral composition in infants of two central African countries (Gabon and Republic of the Congo) during their first month of life

**DOI:** 10.1371/journal.pone.0185569

**Published:** 2017-10-02

**Authors:** Lionel Brazier, Eric Elguero, Claudine Kombila Koumavor, Nicolas Renaud, Franck Prugnolle, Frédéric Thomas, Simon Ategbo, Moyen Engoba, Obengui, Eric M. Leroy, Patrick Durand, François Renaud, Pierre Becquart

**Affiliations:** 1 UMR 5290 MIVEGEC, Institut de Recherche pour le Développement (IRD), Montpellier, France; 2 Centre International de Recherches Médicales de Franceville, Franceville, Gabon; 3 Labco-Midi, Montpellier, France; 4 Centre Hospitalier Universitaire d'Angondjé, Libreville, Gabon; 5 Centre Hospitalier Universitaire de Brazzaville, Brazzaville, Republic of the Congo; Free University of Bozen/Bolzano, ITALY

## Abstract

Few studies have analyzed the gut microbiota of child in unindustrialized countries, but none during the first month of life. Stool samples were collected from healthy newborns in hospitals of Gabon (n = 6) and Republic of the Congo (n = 9) at different time points during the first month of life: meconium, day 2 (D02), day 7 (D07) and day 28 (D28). In addition, one fecal sample was collected from each mother after delivery. Metagenomic sequencing was performed to determine the bacterial communities, and multiplex real-time PCR was used to detect the presence of seven enteric viruses (rotavirus a, adenovirus, norovirus I and II, sapovirus, astrovirus, enterovirus) in these samples. Bacterial diversity was high in the first days of life, and was dominated by the genus *Prevotella*. Then, it rapidly decreased and remained low up to D28 when the gut flora was composed almost exclusively of strictly anaerobic bacteria. Each infant’s fecal bacterial microbiota composition was significantly closer to that of their mother than to that of any other woman in the mothers’ group, suggesting an intrauterine, placental or amniotic fluid origin of such bacteria. Moreover, bacterial communities differed according to the delivery mode. Overall, the bacterial microbiota communities displayed a similar diversification and expansion in newborns within and between countries during the first four weeks of life. Moreover, six of the fifteen infants of this study harbored enteric viruses (rotavirus, enterovirus and adenovirus) in fecal samples, but never in the meconium. A maternal source for the viruses detected at D02 and D07 can be excluded because none of them was found also in the child’s mother. These findings improve our knowledge on the gut bacterial and viral communities of infants from two Sub-Saharan countries during their first month of life.

## Introduction

The microbiota is the community of microorganisms (bacteria, viruses, parasites, fungi) that live in a specific environment. In humans, the microbiota composition varies according to the body site (skin, oral cavity, vagina, gut, or nostril) [1–2]. The human gut microbiota is the most important and is constituted by 10^12^ to 10^14^ microorganisms [3] that represent from 2 to 10 times the number of cells in the whole human body. It is now acknowledged that the gut microbiota plays a role in the digestive, metabolic, immune and neurological functions [4–5]. Consequently, understanding dysbiosis is an important step in the study of some diseases, particularly chronic inflammatory bowel disease [6–7] and autoimmune diseases [8].

Throughout life and particularly at the time of and in the first weeks and months after birth, the gut microbiome (i.e., the microbiota genes and genomes) continues to change. It has been reported that the first founding populations of the digestive tract microbiota are influenced by the delivery mode [9, 10]. Indeed, the gut of infants born by natural delivery is seeded by the mother’s vaginal and fecal bacteria [11–12], with a predominance of *Bifidobacterium* and *Lactobacillus*. Conversely, the gut of newborns delivered by Cesarean section (C-section) is seeded by environmental bacteria that are not necessarily specific to the intestinal microflora [13–14]. Then, the gut microbiome changes during the first year of life until it reaches the composition characteristic of adulthood [12].

Despite the growing number of publications on "gut microbiota" found in the National Center for Biotechnology Information [15] (from 380 items in 2010 to 3,193 in 2016), few studies have been conducted in unindustrialized countries and among populations with traditional lifestyle. To date, three studies focused on the gut microbiome of South American populations [16–18] and four on Asian populations [19–22]. The African continent is also underrepresented in metagenomic studies [16, 23–26]. These studies focused on the difference in microbial diversity in populations with contrasting subsistence modes: hunter-gatherers with traditional subsistence practices [17–18, 24–26] and rural agriculturalists with more western-like subsistence practices [16, 18, 20–23, 25–26].

The question concerning the first bacterial contact of the digestive tract (*i*.*e*. the first element of seeding) is crucial and remains unclear [27–28]. Indeed, despite the broadly agreed consensus that the gestational environment and the fetus are sterile until delivery [29], many studies showed the presence of bacterial DNA in the amniotic fluid [30–31], umbilical cord [32] and placenta [31, 33]. The presence of bacteria in these compartments could explain the transmission of bacteria from mother to fetus and why the meconium, which is composed of material ingested during gestation (intestinal and epithelial cells, lanugo, mucus, amniotic fluid, bile and water), is not germ-free [9, 34]. Moreover, human enteric viruses are frequently detected in infants’ stool samples [35]. Although infections by enteric viruses mainly result in minor or no symptoms in healthy children, they can cause diarrhea [36–37]. Infectious diarrhea is responsible of the death of many children, especially young infants living in developing countries, due to poor hygiene, unsanitary water, contaminated food, or inadequate disposal of waste and feces [38–39].

Therefore, it is important to analyze the different steps involved in the infant gut bacterial/virus seeding and to monitor the changes of the gut microbiota community in the early days of life. To this aim, we collected fecal samples of newborns in two non-industrialized countries, Gabon and Republic of the Congo, during the first month of life to assess their bacterial/viral diversity, as an indication of the gut microbiota profile. We then used these data to try to answer several questions: a) What is the seeding source of the bacteria/viruses present in newborn gut? b) Is the meconium bacterial richness different compared with that of the subsequent fecal samples? c) What are the routes taken by bacteria to colonize the gut during the first month of life? Are there differences in richness and organization of bacteria associations? d) What are the effects of the delivery mode (natural *vs*. C-section) and feeding methods (breastfeeding *vs*. formula feeding) on the bacterial/viral communities? e) Which enteric viruses are present early in life? f) Are the structure and composition of the analyzed microbiomes different between Gabon and Republic of the Congo?

## Materials and methods

### Origin of samples

#### Subjects’ recruitment

The study population included newborns and their mothers who were recruited in the university hospital of Angondjé, Gabon, and the university hospital of Brazzaville, Republic of the Congo. Patients and the medical personnel of the relevant neonatal services were recruited in the study according to the concept of free and voluntary participation and the consent form was signed by a parent/legal guardian and by the health staff. The study was approved by the Gabon National Ethics Committee for Research (internal reference: PROT N°0049/2016/SG/CNE).

#### Stool sample collection

Stool samples (Table 1) from nine Congolese newborns (C1 to C8, including the twins C5.1 and C5.2) and from six Gabonese newborns (G1 to G6) were collected from July to September 2014 and in June-July 2014, respectively. For each newborn, four consecutive fecal samples were collected: meconium, day 2 (D02), day 7 (D07), and day 28 (D28). In addition, one fecal sample was collected from each mother (M1 to M14) after delivery.

**Table 1:** Information on the children included in the study.

Newborn	Country	Delivery mode	Gestational age (weeks of amenorrhea)	Feeding	Sex	Birth weight
C1	RC	vaginal	normal term (37–41.5 wa)	Breast	M	3600 g
C2	RC	vaginal	postterm (> 41.5 wa)	Breast	M	3200 g
C3	RC	vaginal	normal term (37–41.5 wa)	Breast	F	2800 g
C4	RC	C-section	preterm (< 37 wa)	Formula	F	2700 g
C5-1	RC	C-section	preterm (< 37 wa)	Breast	M	2300 g
C5-2	RC	C-section	preterm (< 37 wa)	Breast	M	2100 g
C6	RC	C-section	postterm (> 41.5 wa)	Breast	M	3300 g
C7	RC	C-section	normal term (37–41.5 wa)	Breast	M	3650 g
C8	RC	vaginal	normal term (37–41.5 wa)	Breast	F	2750 g
G1	Gabon	vaginal	postterm (> 41.5 wa)	Mixed	M	3525 g
G2	Gabon	vaginal	normal term (37–41.5 wa)	Breast	M	3680 g
G3	Gabon	C-section	normal term (37–41.5 wa)	Formula	M	3485 g
G4	Gabon	C-section	normal term (37–41.5 wa)	Formula	M	4405 g
G5	Gabon	C-section	normal term (37–41.5 wa)	Mixed	M	3505 g
G6	Gabon	vaginal	normal term (37–41.5 wa)	Mixed	M	3395 g

RC = Republic of the Congo; wa = weeks of amenorrhea; breast = breastfeeding; mixed = breastfeeding + formula feeding; formula = bottle milk; C-section = Cesarean section; M = male; F = female. The mean birth weight of the children included in the study was higher than 2,100g.

Each stool sample was collected non-invasively in a sterile 50 mL tube and stored in RNAlater® (Sigma) at 4°C just after collection and then -20°C until DNA/RNA extraction.

### Bacterial community data acquisition

#### DNA extraction

Total genomic DNA was isolated from 500 μL of filtered stool sample using the QIAamp Fast DNA Stool mini kit (QIAGEN) and following the manufacturer’s instructions. DNA samples were eluted in 200 μL of ATE buffer (QIAGEN) and stored at -20°C. Extraction controls (500 μL of sterile water) were processed in parallel to monitor for contamination.

#### PCR amplification of the V3 region of 16S

The V3 region of the 16S rRNA gene was amplified using the broad range bacterial-specific primers Probio-Uni and Probio-Rev [40]. These primers amplify a fragment of 181 bp that includes more than 94% of the bacterial 16S rRNA coding sequence [40]. Primers included a 10-mer barcode and adaptor sequences needed for the Ion Torrent PGM sequencing technology. PCR amplification was performed as described previously [40] with Q5® DNA Polymerase (New England Biolabs) on an Eppendorf thermal cycler with the following cycling program: 5min of denaturation at 98°C, followed by 25 cycles of 30sec at 98°C, 15sec at 50°C, 15sec at 72°C, with a final extension at 72°C for 10min. To avoid amplification bias as much as possible, each sample was amplified in four separate 20 μL mixtures that were pooled at the end of the amplification. The amplicon quality was checked with the High Sensitivity DNA kit (Agilent Technology) on a 2100 Bioanalyzer instrument (Agilent Technology).

#### Ion torrent PGM 16S metagenomic sequencing

For DNA library construction, PCR products were purified twice by magnetic separation using Agencourt AMPure XP DNA purification beads (Beckman Coulter Genomics). Then, the elimination of free primers and the library yields were verified by electrophoresis on a 2100 Bioanalyzer Agilent apparatus (Agilent Technology). All amplicons were quantified and pooled to equalize their concentrations for emulsion PCR with the Ion One Touch 400 Template Kit v2 (Life Technologies) following the manufacturer’s instructions. After loading on Ion 318™ chips (n = 48 library/chip), the barcoded amplicon libraries were sequenced using the Ion PGM™ Hi-Q™ Sequencing kit.

**Fig 1:**
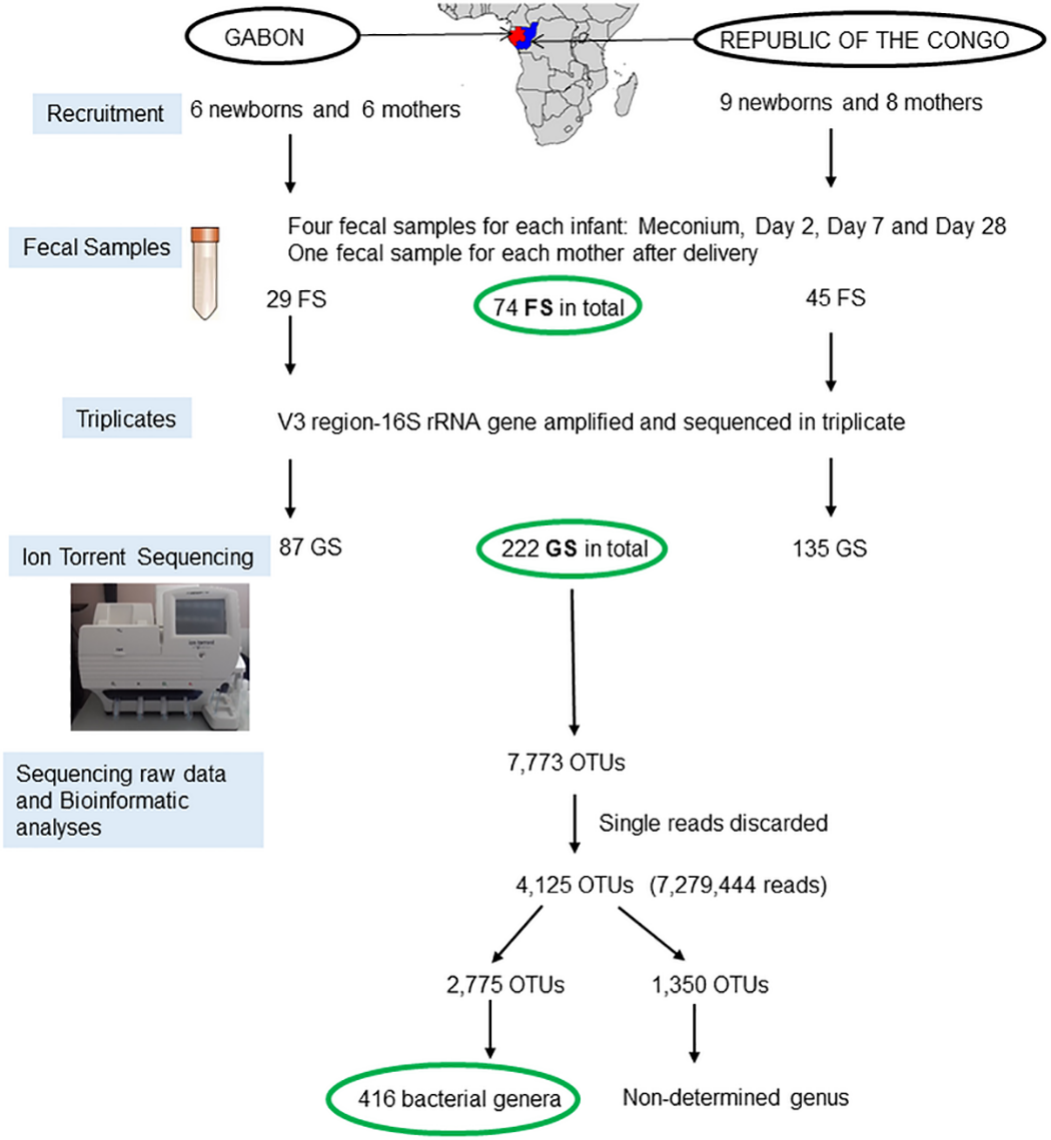
Study location and data acquisition process. OTU: (Operational Taxonomic Unit). African map (source: CIA World Data Bank II; http://www.evl.uic.edu/pape/data/WDB/).

To validate the protocol reliability and repeatability, fecal samples were amplified and sequenced three times separately (i.e. triplicates for each sample). In this study, *fecal samples* define the collected material, whereas *genetic samples* the sequencing results. Thus, for each fecal sample, there were three genetic samples, with few exceptions. Infant C3 had only two genetic samples of meconium; infant G4 had two genetic samples for the D7 fecal sample, and infants G2 and G6 had four genetic samples for meconium. In all, there were 74 fecal samples (60 for the infant group and 14 for the mother group) and 222 genetic samples. The data acquisition process is summarized in Fig 1.

To monitor reagent contamination by bacterial DNA [41], four negative controls (500μl sterile water instead of DNA) were processed for each DNA extraction and they were sequenced at the same time as the samples.

### Bacterial metagenomic analysis

The standard flowgram format (sff) files (raw data) for each sample of each chip were downloaded from the Torrent Server and processed using Mothur v1.35.1 [42]. The standard operating procedure (SOP) for 454 pyrosequencing [43] was adapted to the Ion Torrent technology. Briefly, sequences were extracted from the sff files and converted into fasta format. Then, they were filtered to keep only sequences with a) homopolymers of 8 bases maximum, b) a mean rolling quality window ≥30bp, c) a quality window size of 50 bases, d) no more than one mismatch in the primers and e) no more than one mismatch in the barcode. All the.fasta and.qual files were concatenated in one single file that was used for: a) sequence alignment using Silva SSU Reference alignment v102 [44]; b) removal of sequencing errors and chimeric sequences with the Uchime program [45]; c) sequence assignment using the method described by Wang *et al*. [46] and a bootstrap value of 80; d) sequence clustering in Operational Taxonomic Units (OTUs) with 97% sequence homology; e) filtering and removal of OTUs represented by a single sequence; and f) the consensus taxonomic classification of each OTU (up to the genus).

To assess the repeatability of the genetic data acquisition process, each mother/child pair was studied separately (four fecal samples from the infant and one from the mother and thus, 15 genetic samples in total with triplicates). For each of the 15 genetic samples an OTU frequency profile was computed. Pairwise Chi-squared distances between OTU frequency profiles were computed and a single-linkage clustering algorithm was used to build a tree. The repeatability was considered correct for a given fecal sample when its three genetic samples formed a separate cluster.

### Bacteria data analysis

As most OTUs were attributed to a bacterial genus, genera were used to study ecological questions, and the more discriminating OTU classification to study the possible mother to newborn transmission.

#### Mother/child relationship

To study the relationship between the child and mother fecal microbiota, for each pair of samples the number of shared OTUs was computed, and permutation tests were performed to determine whether the child's samples shared more OTUs with the mother's sample than with any another randomly selected woman in the mothers’ group. The average number *d*_0_ of shared OTUs between child and mother was computed. Then, mothers were randomly attributed to children and the average number of shared OTUs was computed. This process was repeated 10,000 times, to produce a sequence *d*_1_,…,*d*_10000_. The test P-value was taken as the proportion of *d*_i_ above the observed value *d*_0_. Infant samples at different time points were studied separately.

#### Bacterial diversity changes

To study the establishment of the gut bacterial community, bacterial genera were considered. The Shannon diversity index and the generic richness were computed and plotted against the sampling time.

#### Bacterial microbiota and individual characteristics

To study the influence of country, delivery mode and feeding method on the bacterial microbiota, bacterial genera were investigated using linear mixed models with a random term corresponding to the child. As several bacterial genera were present only in one or two children, thus making the fitting of a linear model impossible or meaningless, rare genera were discarded and the analysis focused on bacterial genera with a read frequency higher than 5% in at least one child. The Benjamini-Hochberg correction [47] was used to control the false discovery rate. All computations were performed with the R software [48] and specifically the lme4 package [49].

### Viral data acquisition

#### Nucleic acid extraction

Fecal samples were centrifuged at 1,500 rpm for 5 min to remove the RNAlater® solution and were resuspended in phosphate buffered saline. Supernatants were either stored at –80°C or used immediately for DNA and RNA extraction with the QIAamp DNA Mini Kit (Qiagen) and QIAamp Viral RNA Mini Kit (Qiagen), respectively.

#### Virus detection and genotyping

To determine the extent of viral infections occurring early in life, the presence of six RNA virus groups (Astrovirus, Norovirus I and II, Sapovirus, Enterovirus and group A Rotavirus) and one DNA virus group (Adenovirus) was assessed by multiplex real-time PCR or reverse transcription (RT)-PCR, using previously published specific primers, probes and conditions [50–53]. All PCR amplifications were performed in a 7500 Real Time PCR System (Applied Biosystems), with one positive control and three negative controls, kindly provided by the French National Reference Center for Gastroenteritis Viruses (Dijon, France).

In the case of positive results, genotyping was performed as described elsewhere [54–59] using the controls described above. Positive PCR products were sequenced. Raw sequences were edited and manually corrected with Geneious R7. Neighbor-joining trees and genetic distances were calculated using the MEGA7 software with the Tamara 3-parameter G/Gamma distributed with invariant sites.

### Virus statistical analysis

A logistic regression model was employed to study the association between infection by a given virus and country, delivery mode and feeding method. To study the association between viral and bacterial presence in the samples, mixed linear models were fitted with the logarithm-transformed frequencies for each bacterial genus as response and the presence/absence of a virus as explaining variable. In all cases, a random effect was added to take into account the grouping of samples from the same child.

## Results

### Bacteria

#### Bioinformatics results

The 222 genetic samples produced 7,773 OTUs. OTUs described by a single read were discarded because they were not representative of a community. Finally, 4,125 OTUs and 7,279,444 reads were retained.

The four negative controls, which were handled (from DNA extraction to OTU attribution) as and concomitantly with the samples, contained 30,228 reads (0.4% of the total). For each OTU in the dataset, the maximum number of reads in any negative control was determined and the dataset was trimmed using this threshold. For example, as OTU19 yielded 5,937 reads in one of the negative controls, 5,937 reads were subtracted from all sample counts for this OTU (possibly leading to the complete removal of the OTU in some samples).

When one of the three genetic samples for each fecal sample did not form a cluster with the other two, the sample was discarded. Supporting Information S1 Fig exemplifies this procedure.

In only 10 (meconium of children C1, C2, C3, C4, C5.1 and G3; D02 sample of children C2, G3; D07 sample of child G3 and D28 sample of child C2) of the 74 fecal samples, the relevant genetic samples did not form a separate cluster. This indicates that the process repeatability was correct for 86% (64/74) of fecal samples. Then, the genetic samples pertaining to the same fecal sample were pooled to produce the final dataset. Overall, the excluded genetic samples had an average of 18,606 reads, compared with 32,949 reads for the retained samples.

From now on, *sample* designates the pooled genetic data corresponding to a single fecal sample. Thus, the final dataset contained 74 genetic samples from 15 children. These samples included 6,985,225 reads and 3,968 OTUs.

#### Genus attribution

Among the 3,968 OTUs, 70% (2,775) were attributed to 416 bacterial genera. Conversely, 30% (1,193 OTUs) could not be attributed to a genus because they did not match any sequence in the reference database, or they matched a group with uncertain taxonomical status. The 2,775 OTUs with known genus represented 92% of all reads (n = 6,439,348).

#### Does the meconium contain bacteria?

Table 2 shows that meconium samples contain numbers of reads and OTU comparable to those in the other infant samples. Specifically, 296 of the 416 identified bacterial genera were present in meconium samples, and the most frequent genera were: *Prevotella* (20%), *Enterococcus* (11%), *Streptococcus* (9%), *Acinetobacter* (8%), and *Bradyrhizobium* (8%). Moreover, 87 bacterial genera were present only in meconium samples, particularly *Amphibacillus* (4,602 reads), *Herbaspirillum* (414 reads), *Ornithinicoccus* (256 reads), *Segetibacter* (260 reads) and *Jeotgalicoccus* (211 reads). These last genera are typical environmental bacteria (soil, plants, lakes, marine mud) and were not present in the negative controls.

**Table 2:** Averages and ranges of the reads numbers and OTU numbers for mothers, meconium, D02, D07 and D28.

Fecal sample	Reads number average	Reads number range	OTU number average	OTU number range
mothers	144,056	34,446–490,413	573	60–1,352
meconium	66,039	5,127–257,420	154	42–295
D02	59,797	11,106–133,672	114	34–334
D07	66,443	31,613–156,080	84	46–171
D28	138,950	21,309–322,634	109	37–196

OTU: Operational Taxonomic Unit.

#### Are the child and mother’s bacterial microbiota similar?

Permutation tests showed that at all sampling time points, the bacterial composition of a child sample was more similar to the mother’s sample than to the sample of any other woman in the mothers’ group. The child-mother similarity tended to increase during the first month of life. Indeed, the meconium sample shared on average 12% more OTUs and the D28 sample 21% more OTUs with the mother’s sample than with that of any other woman (50.6 *vs*. 45 and 58.7 *vs*. 48.7 shared OTUs, respectively). Table 3 summarizes these results.

**Table 3:** Results of the permutation tests. Average numbers of shared OTUs between the newborn samples and the sample of the mother or of the other women included in the mothers’ group.

Fecal sample	Mean number of OTUs shared with mother	Mean number of OTUs shared with other women	P-value
meconium	50.6	45.0	0.030
D02	54.8	48.7	0.0043
D07	44.1	37.9	0.0001
D28	58.7	48.7	<1E-7

#### How does the fecal bacterial community change during the first month of life?

**Fig 2:**
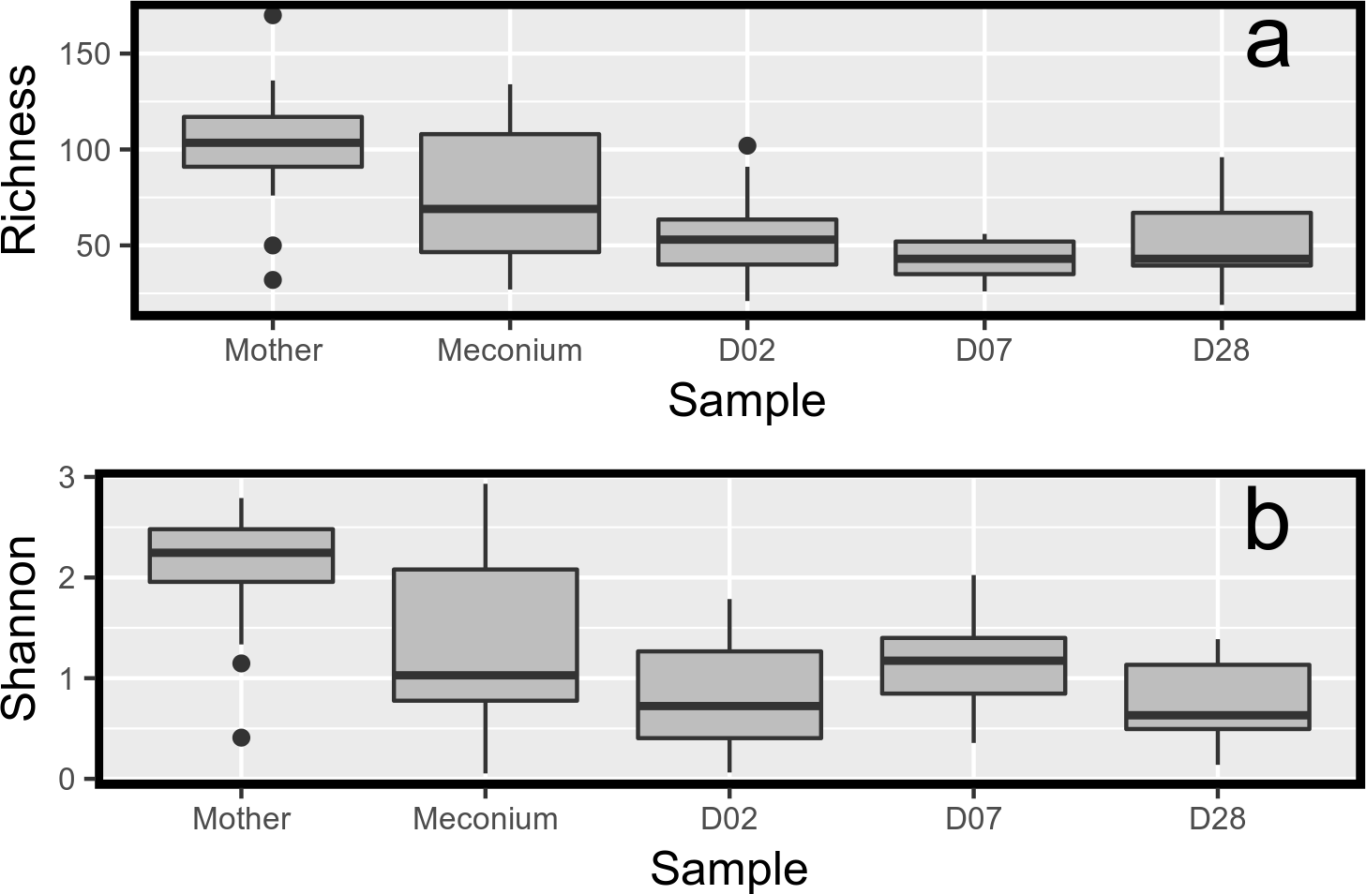
Diversity index changes. Diversity index changes during the first month of life and between mothers and infants. a: Generic richness. b: Shannon diversity index.

The distribution of the individual generic richness (Fig 2A) and Shannon index (Fig 2B) showed that the bacterial microbiota diversity was much larger in the mothers than in the children at any time point during the first month of life. The child bacterial richness values were more similar to those of the mother for the meconium samples, despite their high variability, but they rapidly decreased from D02. In agreement, when samples from a given time point were pooled to calculate the generic richness, 263 different genera were found in the mothers’ samples, 296 in meconium, 194 in D02, 132 in D07 and 174 in D28 samples.

**Fig 3:**
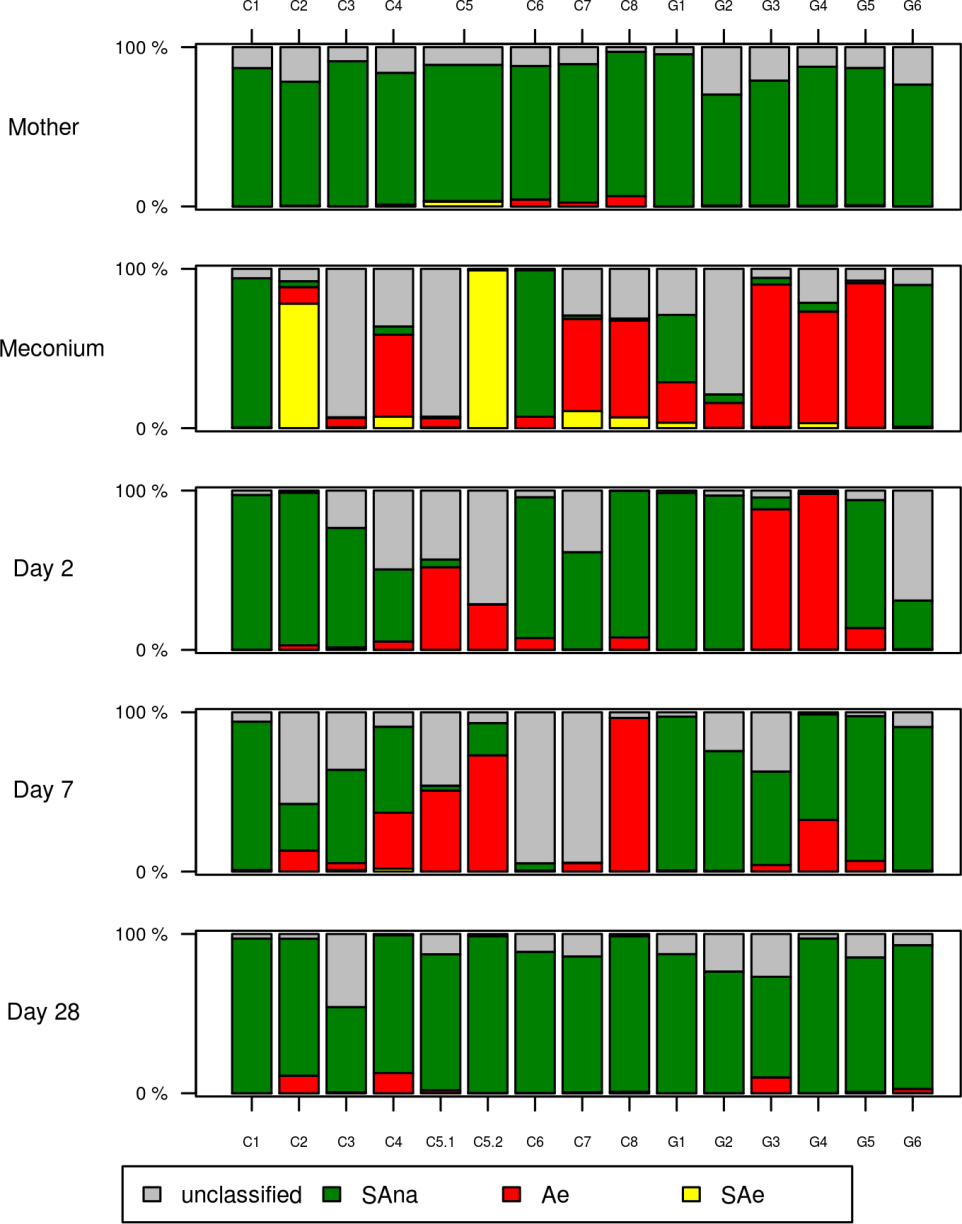
Relative abundances of aerobic or facultative anaerobic (Ae, red), strictly aerobic (SAe, yellow) and strictly anaerobic (SAna, green) bacteria in all samples. Grey areas represent the relative abundances of OTUs not attributed to a genus or attributed to a genus whose respiratory mode is unknown.

To further investigate this question, the bacterial genera were classified as aerobic or facultative anaerobic (Ae), strictly aerobic (SAe) and strictly anaerobic (SAna) and the proportion of each group was determined in individual samples at the different time points (Fig 3). The bacterial community of all mothers’ samples was essentially composed of strictly anaerobic bacteria. Although in all children, strictly anaerobic bacteria were prevalent at D28, the establishment of this flora composition showed some temporal variations (from D02 in some children to D28 in others).

#### Is the fecal bacterial composition influenced by the country, delivery mode and feeding method?

In terms of diversity (Shannon index) and generic richness, there were no significant differences according to either country, delivery mode or feeding method.

**Fig 4:**
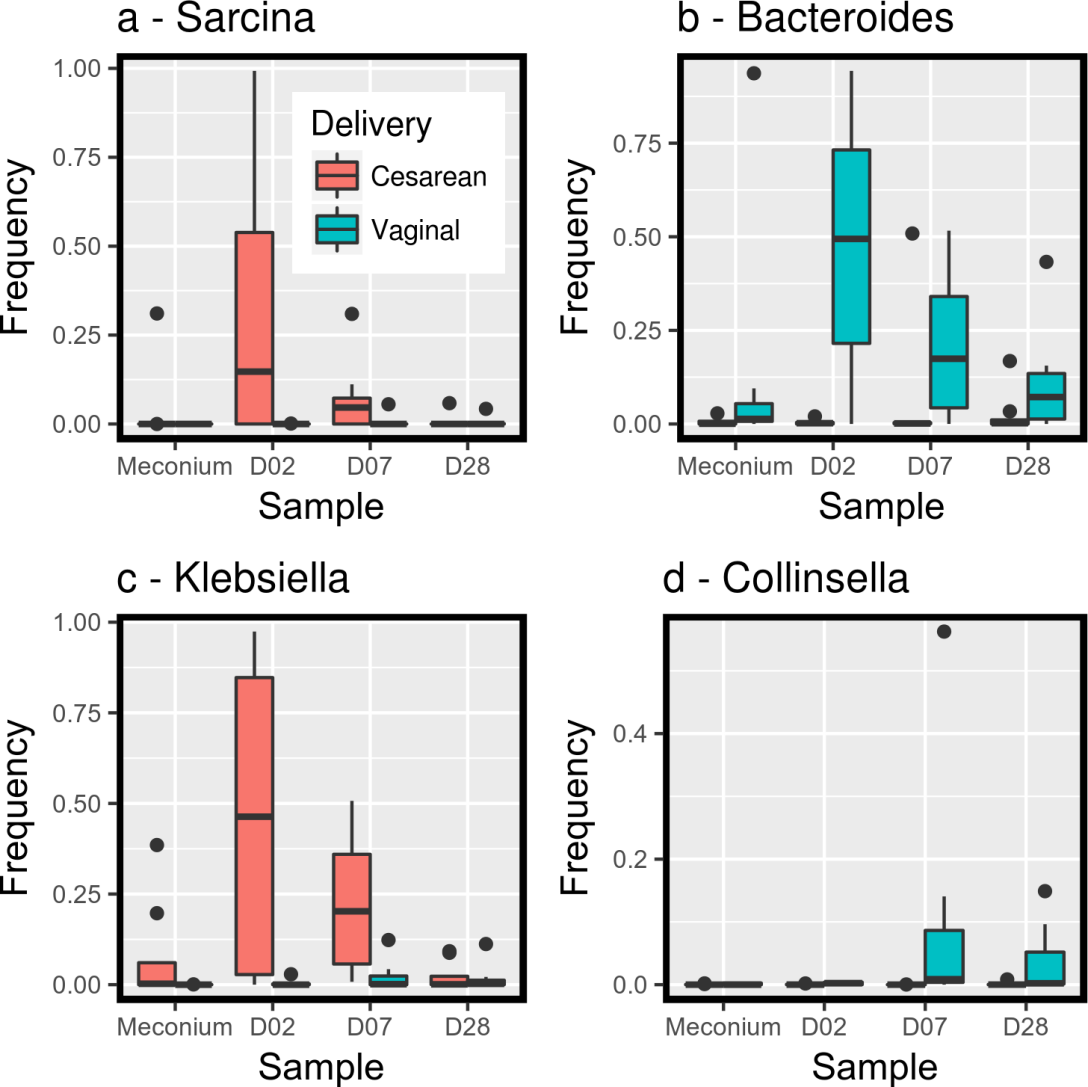
Relative abundance of four bacterial genera in the infants’ samples according to the sampling day and delivery mode (Cesarean sections versus vaginal delivery). a: *Sarcina* b: *Bacteroidetes* c: *Klebsiella* d: *Collinsella*.

**Fig 5:**
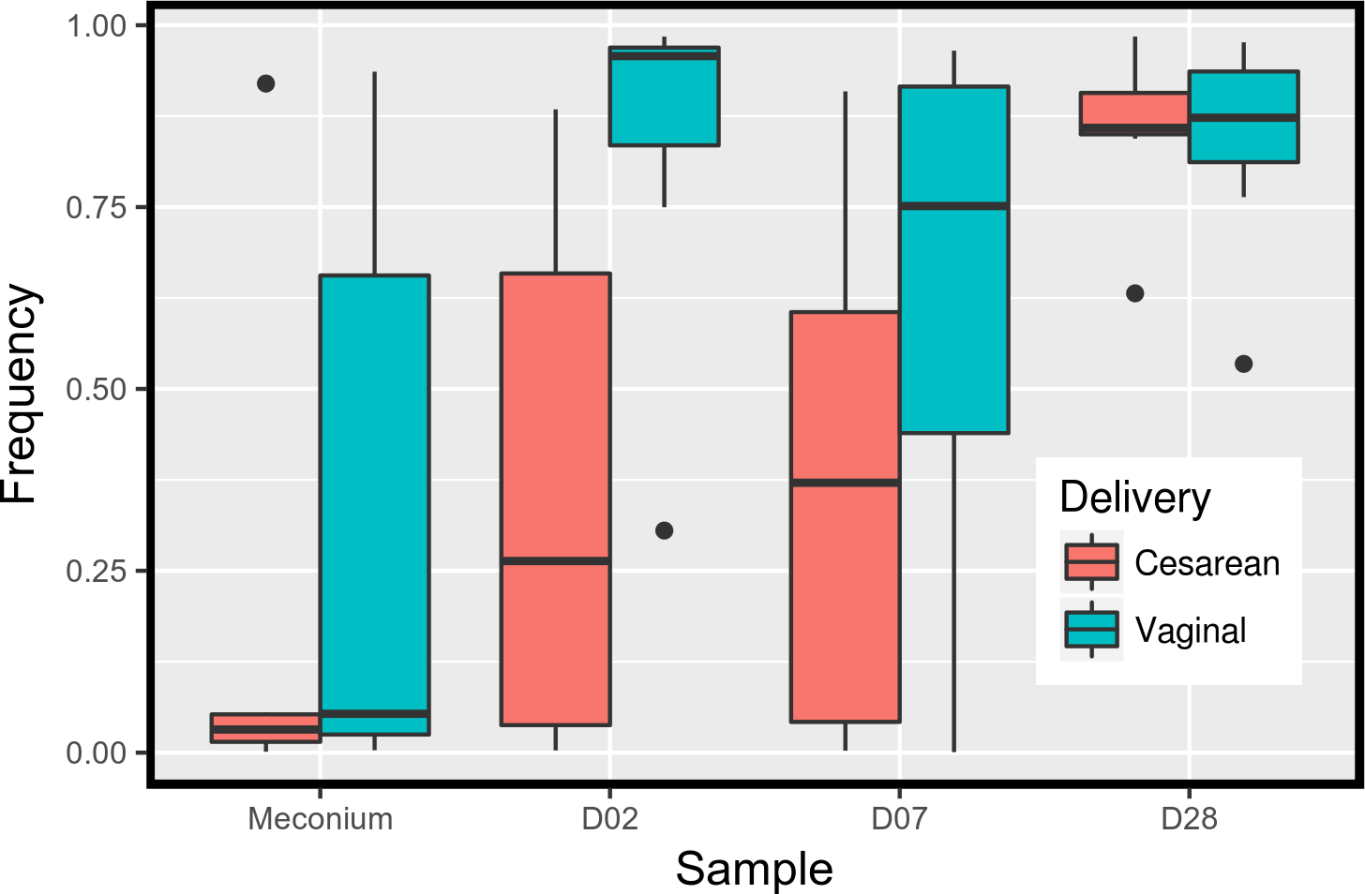
Relative abundance of strictly anaerobic bacteria in the infants’ samples according to the sampling day and delivery mode (Cesarean sections versus vaginal delivery).

In a genus by genus approach, no significant difference was found between these Central African countries with different public health policies and also between feeding methods. Conversely, the delivery mode influenced the sample composition. After eliminating the rare bacterial genera (less than 5% in all samples), among the remaining 37 genera, *Bacteroides* (P-value = 0.0023) and *Collinsella* (P-value = 0.0042) were significantly more abundant in samples of children born by vaginal delivery, and *Klebsiella*, (P-value = 0.0011) and *Sarcina* (P-value = 0.0028) in samples of children born by C-section at all different time points (Fig 4). Similarly, when bacteria were classified in aerobic and anaerobic, the relative abundance of strictly anaerobic bacteria was strongly associated with the delivery mode (P-value = 0.023) (Fig 5).

### Enteric viruses

At least one enteric virus was detected in two of the six infants from Gabon (33.3%) and in four of the nine infants from Republic of the Congo (44.4%). All the viruses detected in the infants’ samples were never found in the respective mother’s sample.

Viruses were never detected in the meconium samples. Rotavirus A was detected in one child (G1 D02) (16.7%) and in one mother (M3) from Gabon and in four children (44.4%) from Republic of the Congo. The virus was detected in two consecutive samples in three of the four children from Republic of the Congo (D02 and D07 for C5.2, C6; D07 and D28 for C7) and in only one sample (D28) for C4 (S1 Table). Adenovirus was detected in one child (16.7%) from Gabon (G3 at D02 and D07) and in two children (22.2%) from Republic of the Congo (C4 at D07 and D28; C6 at D02 and D07) (S1 Table). Enterovirus was found in one child (G4) (16.7%) from Gabon and five children (C1, C2, C3, C7 and C8) (55.6%) from Republic of the Congo. Norovirus 1 and 2 and astrovirus were never detected by real time PCR.

Among the rotavirus-positive samples, one sample was successfully genotyped (C4 at D28 in S1 Table) and clustered with the Rotavirus A strain G1 (S2 Fig). Moreover, the sequences of four adenovirus-positive specimens (G3 at D02 and D07; C4 at D28; C6 at D02) were also determined and they all clustered with the human adenovirus (HAdV) type F serotype 41 strain (S2 Fig). No mutation of HAdv-41 sequence was found in the only child (G3) where two HAdv-positive samples (D02 and D07) were genotyped. After sequencing, enteroviruses present in samples were characterized as polioviruses.

Virus detection was not significantly different between countries, birth modes and feeding methods. No significant association was found between the abundance of bacterial genera and the presence of viruses.

## Discussion

### Bacterial diversity of meconium

Our study shows that bacterial diversity is high in the meconium, but then rapidly decreases at D02 and stays low up to D28. This suggests that bacterial colonization starts before birth, challenging the traditional dogma of uterine sterility [33]. By comparing the bacterial diversity of meconium and adult fecal samples, Hu *et al*. [60] found a lower bacterial diversity in the meconium; however, their study concerned mothers with diabetes that could have influenced the maternal gut microbiota composition. Interestingly, the most abundant genus in meconium is *Prevotella* that is associated with plant-rich diets (high intake of complex carbohydrates and fruits and vegetables). De Filippo *et al*. [23] found that compared with European children, the fecal microbiota of 1-6-year-old children from a rural village of Burkina Faso was significant enriched in Bacteroidetes and poor in Firmicutes. Moreover, bacteria from the *Prevotella* genus were particularly abundant in the African samples, whereas they were absent in the European samples. Similarly, Gomez *et al*. [26] reported that *Prevotella* genus bacteria were abundant in samples from adult BaAka hunter-gatherers from Central African Republic, while undetectable in US Americans. Ardisson *et al*. [61] also did not find *Prevotella* genus bacteria in meconium samples from 52 US infants. Conversely, Nagpal *et al*. [62], who studied 151 healthy full-term Japanese infants, noted the presence of *Prevotella* in only 1.5% of meconium from vaginally born infants, but never from those delivered by C-section. Therefore, this is the first time that the presence of *Prevotella* is reported in such a high proportion in meconium, which suggests that Prevotella is probably in the vaginal commensal flora. The presence of environmental bacteria in meconium could be caused by a possible contamination at the time of sample collection.

### Maternal impact on the bacterial colonization process

Our study shows that each infant’s bacterial microbiota is significantly closer to that of the mother than to that of any other woman in the mothers’ group, suggesting that the mother is actively involved in the initial seeding of her child’s gut bacterial microbiota. This is in agreement with studies on *in utero* transmission of the first bacterial populations. Jimenez *et al*. [34] demonstrated in an experimental murine model the transmission of labeled strains from the digestive tract of a pregnant female to its offspring meconium. Based on this previous finding and on their result that the dominant bacterial genera in the meconium are different from those found in the fecal, skin or vaginal environments of pregnant women, Gosalbes *et al*. [63] suggested that the meconium bacterial microbiota has an intrauterine origin. Placenta [33] and amniotic fluid [31] could be other sources of the meconium bacterial microbiota. Therefore, the meconium microbial composition could reflect the *in utero* environment where microbes are transmitted from the mother to the fetus when swallowing amniotic fluid. This could also explain the larger diversity observed in the meconium bacterial microbiota composition, because the bacteria present *in utero* can be quite different between mothers.

The bacterial microbiota richness of the fecal samples (D02 to D28) is lower than that of the mothers’ samples. This could be explained by age-related physiological differences, but also by the diet diversity, because infants are essentially fed milk (i.e., maternal or formula).

### Effect of the delivery mode on bacterial communities

Our findings indicate that the *Bacteroides* and *Collinsella* genera are more abundant in the fecal bacterial microbiota of children born naturally, while the *Sarcina* and *Klebsiella* genera are predominant in children born by C–section. Species of the genus *Klebsiella* are present worldwide, but particularly in tropical and subtropical regions. *Klebsiella* species show high colonization rates in newborns [64] and this genus is considered to be endemic in hospital where nosocomial infections are frequent. Moreover, several *Bacteroides* species are involved in the metabolism of human milk [65]. Therefore, *Bacteroides* depletion in infants born by C-section could lead to problems in milk digestion. This issue could be more acute in countries with limited resources for public health care.

Azad *et al*. [66] and Penders *et al*. [67] showed the underrepresentation of *Bacteroides* in cesarean-section delivered children. Along the same lines, a recent review by Rutayisire *et al*. [68] showed that the delivery mode affects the diversity and colonization pattern of the gut bacterial microbiota during the first three months of life. Other studie [69] did not find any effect of the delivery mode on the gut bacterial microbiota and suggested that the composition of the early infant gut bacterial microbiota is only associated with the maternal diet during the last three months of pregnancy.

### Establishment of the anaerobic bacterial flora

In D28 samples, the gut flora was composed almost exclusively of strictly anaerobic bacteria. In these samples, the *Bifidobacterium* genus represented 74.5% (1,367,569/1,835,716) of all reads of strictly anaerobic bacteria. *Bifidobacterium* bacteria from the mother facilitate the assimilation of breast milk by the child, but also the use oligosaccharides present in breast milk as prebiotic [70]. These sugars are quantitatively one of the main constituents of breast milk and are partially digested in the small intestine before reaching the colon where they stimulate the development and growth of bifidogenic flora [70]. This specific flora has the main effect of initiating the education of the newborn's immune system [10].

In a large US population-based cohort of infants, it was shown that during the first six weeks of life the neonatal bacterial microbiota is significantly reorganized with the appearance of the communities present in the adult [71]. In our study, we observed that the proportion of strictly anaerobic bacteria increased rapidly and represented the majority of the microbial community at D28. We did not find significant differences in microbial richness and organization between Gabon and Republic of the Congo. This could indicate that the same changes in the routes of colonization and bacterial composition occur in infants during their first days of life (e.g., a possible “evolutionary homeostasis” process for the gut bacterial microbiota colonization). This was underlined by a previous study proposing that the gross reorganization of the infant bacterial microbiota within the first weeks of life could be generalized across populations [69].

### Fecal viral shedding

In this study, we used classical approaches to detect the most common and widespread human enteric viruses (Adenovirus, Sapovirus, Rotavirus, Norovirus, Astrovirus, and Enterovirus) because they allow detecting in infant and adult feces the main viral communities [72]. The presence of enteric viruses in stool samples in healthy infants has been reported during early childhood (>3 months) [73]. Here, for the first time, the availability of longitudinally collected fecal samples from Gabon and Republic of the Congo allowed the temporal analysis of fecal viral shedding during the first month of life. No enteric virus was found in the meconium, consistent with the literature [35]. Rotavirus, enterovirus and adenovirus were the first viruses detected from D02. Poliovirus presence in the feces is explained by OPV administration. Gabonese children receive the first dose of the trivalent Oral Poliovirus Vaccine (OPV), which contains the three serotypes, at birth or the next day, as prescribed by the expanded immunization program of Gabon. Similarly, the first dose of the bivalent OPV, which contains serotypes 1 and 3, is administered approximately three days after birth in Brazzaville. This may explain the presence of the three poliovirus serotypes in some samples between D02 and D28, as previously reported by Kanungo *et al*. [74] in vaccinated Indian infants. Conversely, the initial source of the other viruses in the infant human gut is unknown, although inoculation from nosocomial, dietary and environmental sources is possible. The maternal source can be excluded because none of the viruses detected at D02 or D07 in newborns was found in the respective mother. Only one mother (M3) was infected by rotavirus, but the virus was never detected in her infant.

Besides poliovirus, rotavirus was the most commonly detected virus from D02 in newborns (33.3%; 5/15). Due to the small number of virus detected in the study, its prevalence was not significantly different between Republic of the Congo and Gabon. However, rotavirus was found mainly in children from Republic of the Congo (44.4% [4/9] *vs*. 20.0% [1/5] from Gabon). Only one rotavirus was genotyped, possibly due to the low viral load, as suggested by the cycle of quantification values higher than 35 for most of the samples screened by real time RT-PCR. All fecal samples from Republic of Congo were collected during the dry season (from June to September) when the rotavirus genotype G1 circulates in the country [75]. Moreover, the virus was found in two consecutive samples in three infants and in one single sample in one child, consistent with the infection short duration [76–77].

HAdv-41 was the second most frequently detected virus (20% [3/15]). Although HAdv-41 is one the main agents of viral diarrhea, especially in children [78–80], it is also common in asymptomatic individuals, especially in young children [78, 81]. Like for rotavirus, no significant prevalence difference was found between Republic of the Congo and Gabon.

## Conclusions

We focused on the bacterial/viral colonization of the human gut in infants from two Sub-Saharan countries, Gabon and the Republic of the Congo. The originality of this study is that fecal samples were collected at different time points during the first month of life and meconium and mothers’ fecal samples were also included. Our results confirm the greater diversity and richness of gut bacterial microbiota in populations from African countries (Malawi [16], Tanzania [24], Cameroon [25], Central African Republic [26]) compared with people from Western countries.

To our knowledge this is the first study focusing on the changes in fecal bacterial/viral diversity and organization in early life (i.e., first four weeks after birth) in two African countries. Indeed, previous works concerned older children [16, 23–26] and compared African rural communities and industrialized communities from Europe and North America. These studies revealed specific gut bacterial microbiota adaptations to the respective lifestyles. Here, we found that overall, by four weeks of age, the infants’ bacterial microbiota communities display a similar diversification and expansion in two neighboring African countries.

## Supporting information

S1 Table Enteric virus detection. PCR and RT-PCR results in fecal samples of 15 infants and their mothers from Gabon and Republic of the Congo. Yellow: positive RT-PCR results, Brown: positives RT-PCR and PCR results. Gray: negative results.
(TIF)

S1 Fig Two examples of trees to illustrate the quality assessment method and exclusion of less reliable data. **a**) Clustering of the genetic samples of child C8 (meconium, D02, Do7 and D28). At all sampling time points, all three genetic samples corresponding to the same fecal sample were in the same cluster. **b**) Clustering of the genetic samples of child C1 (meconium, D02, Do7 and D28). One of the three meconium genetic samples did not cluster with the other two meconium genetic samples. This genetic sample was discarded.
Mec = meconium; Mom = mother; D02, D07, D28 = day 2, 7, 28; 1, 2, 3 = same genetic sample in triplicate.
(TIF)

S2 Fig **Rotavirus (a) and adenovirus (b) phylogenetic trees.** Only bootstrap values ≥60 are shown (500 replicates). Sequences from this work are shown in red and reference sequences are described by their accession numbers and country of origin.
(TIF)

## References

[1] Costello EK, Lauber CL, Hamady M, Fierer N, Gordon JI, Knight R. Bacterial Community Variation in Human Body Habitats Across Space and Time. Science. 2009 12 18;326(5960):1694–7. doi: 10.1126/science.1177486 19892944 10.1126/science.1177486 PMC3602444

[2] Cho I, Blaser MJ. The human microbiome: at the interface of health and disease. Nature Reviews Genetics. 2012 3 13; http://www.nature.com/doifinder/10.1038/nrg3182 10.1038/nrg3182 PMC3418802 22411464

[3] Gill SR, Pop M, DeBoy RT, Eckburg PB, Turnbaugh PJ, Samuel BS, et al Metagenomic analysis of the human distal gut microbiome. Science. 2006;312(5778):1355–1359. doi: 10.1126/science.1124234 16741115 10.1126/science.1124234 PMC3027896

[4] Clemente JC, Ursell LK, Parfrey LW, Knight R. The Impact of the Gut Microbiota on Human Health: An Integrative View. Cell. 2012 3;148(6):1258–70. doi: 10.1016/j.cell.2012.01.035 22424233 10.1016/j.cell.2012.01.035 PMC5050011

[5] Dinan TG, Cryan JF. The impact of gut microbiota on brain and behaviour: implications for psychiatry. Curr Opin Clin Nutr Metab Care. 2015 11;18(6):552–8. doi: 10.1097/MCO.0000000000000221 26372511 10.1097/MCO.0000000000000221

[6] Seksik P, Rigottier-Gois L, Gramet G, Sutren M, Pochart P, Marteau P, et al Alterations of the dominant faecal bacterial groups in patients with Crohn’s disease of the colon. Gut. 2003 2;52(2):237–42. 12524406 10.1136/gut.52.2.237 PMC1774977

[7] Ott SJ. Reduction in diversity of the colonic mucosa associated bacterial microflora in patients with active inflammatory bowel disease. Gut. 2004 5 1;53(5):685–93. doi: 10.1136/gut.2003.025403 15082587 10.1136/gut.2003.025403 PMC1774050

[8] De Palma G, Nadal I, Medina M, Donat E, Ribes-Koninckx C, Calabuig M, et al Intestinal dysbiosis and reduced immunoglobulin-coated bacteria associated with coeliac disease in children. BMC microbiology. 2010;10(1):63. 20181275 10.1186/1471-2180-10-63 PMC2843610

[9] Dominguez-Bello MG, Costello EK, Contreras M, Magris M, Hidalgo G, Fierer N, et al Delivery mode shapes the acquisition and structure of the initial microbiota across multiple body habitats in newborns. Proc Natl Acad Sci U S A. 2010 6 29;107(26):11971–5. doi: 10.1073/pnas.1002601107 20566857 10.1073/pnas.1002601107 PMC2900693

[10] Bäckhed F, Roswall J, Peng Y, Feng Q, Jia H, Kovatcheva-Datchary P, et al Dynamics and Stabilization of the Human Gut Microbiome during the First Year of Life. Cell Host & Microbe. 2015 5;17(5):690–703. 25974306 10.1016/j.chom.2015.04.004

[11] Bettelheim KA, Breadon A, Faiers MC, O’Farrell SM, Shooter RA. The origin of O serotypes of Escherichia coli in babies after normal delivery. J Hyg (Lond). 1974 2;72(1):67–70. 4593741 10.1017/s0022172400023226 PMC2130250

[12] Palmer C, Bik EM, DiGiulio DB, Relman DA, Brown PO. Development of the human infant intestinal microbiota. PLoS biol. 2007;5(7):e177 doi: 10.1371/journal.pbio.0050177 17594176 10.1371/journal.pbio.0050177 PMC1896187

[13] Lennox-King S, O’Farrell S, Bettelheim K, Shooter R. Escherichia coli isolated from babies delivered by caesarean section and their environment. Infection. 1976 9 1;4(3):139–45. 789250 10.1007/BF01638940

[14] Grönlund MM, Lehtonen OP, Eerola E, Kero P. Fecal microflora in healthy infants born by different methods of delivery: permanent changes in intestinal flora after cesarean delivery. J Pediatr Gastroenterol Nutr. 1999 1;28(1):19–25. 9890463 10.1097/00005176-199901000-00007

[15] NCBI Resource Coordinators. Database resources of the National Center for Biotechnology Information. Nucleic Acids Res. 2016 1 4;44(Database issue):D7–19. doi: 10.1093/nar/gkv1290 26615191 10.1093/nar/gkv1290 PMC4702911

[16] Yatsunenko T, Rey FE, Manary MJ, Trehan I, Dominguez-Bello MG, Contreras M, et al Human gut microbiome viewed across age and geography. Nature. 2012 5 9; http://www.nature.com/doifinder/10.1038/nature11053 10.1038/nature11053 PMC3376388 22699611

[17] Clemente JC, Pehrsson EC, Blaser MJ, Sandhu K, Gao Z, Wang B, et al The microbiome of uncontacted Amerindians. Sci Adv. 2015 4 17;1(3). 10.1126/sciadv.1500183 PMC4517851 26229982

[18] Obregon-Tito AJ, Tito RY, Metcalf J, Sankaranarayanan K, Clemente JC, Ursell LK, et al Subsistence strategies in traditional societies distinguish gut microbiomes. Nat Commun. 2015 3 25;6. 10.1038/ncomms7505 PMC4386023 25807110

[19] Lin A, Bik EM, Costello EK, Dethlefsen L, Haque R, Relman DA, et al Distinct Distal Gut Microbiome Diversity and Composition in Healthy Children from Bangladesh and the United States. PLoS One. 2013 1 22;8(1). 10.1371/journal.pone.0053838 PMC3551965 23349750

[20] Zhang J, Guo Z, Lim AAQ, Zheng Y, Koh EY, Ho D, et al Mongolians core gut microbiota and its correlation with seasonal dietary changes. Sci Rep. 2014 5 16;4:5001 doi: 10.1038/srep05001 24833488 10.1038/srep05001 PMC4023135

[21] Dehingia M, Thangjam devi K, Talukdar NC, Talukdar R, Reddy N, Mande SS, et al Gut bacterial diversity of the tribes of India and comparison with the worldwide data. Scientific Reports. 2015 Décembre;5:18563 doi: 10.1038/srep18563 26689136 10.1038/srep18563 PMC4686986

[22] Martínez I, Stegen JC, Maldonado-Gómez MX, Eren AM, Siba PM, Greenhill AR, et al The Gut Microbiota of Rural Papua New Guineans: Composition, Diversity Patterns, and Ecological Processes. Cell Reports. 2015 4; 11(4):527–38. doi: 10.1016/j.celrep.2015.03.049 25892234 10.1016/j.celrep.2015.03.049

[23] De Filippo C, Cavalieri D, Di Paola M, Ramazzotti M, Poullet JB, Massart S, et al Impact of diet in shaping gut microbiota revealed by a comparative study in children from Europe and rural Africa. Proceedings of the National Academy of Sciences. 2010; 107(33):14691–14696. 10.1073/pnas.1005963107 PMC2930426 20679230

[24] Schnorr SL, Candela M, Rampelli S, Centanni M, Consolandi C, Basaglia G, et al Gut microbiome of the Hadza hunter-gatherers. Nature Communications. 2014 4 15; 5. 10.1038/ncomms4654 PMC3996546 24736369

[25] Morton ER, Lynch J, Froment A, Lafosse S, Heyer E, Przeworski M, et al Variation in rural African gut microbiota is strongly correlated with colonization by Entamoeba and subsistence. PLoS Genet. 2015; 11(11):e1005658 doi: 10.1371/journal.pgen.1005658 26619199 10.1371/journal.pgen.1005658 PMC4664238

[26] Gomez A, Petrzelkova KJ, Burns MB, Yeoman CJ, Amato KR, Vlckova K, et al Gut Microbiome of Coexisting BaAka Pygmies and Bantu Reflects Gradients of Traditional Subsistence Patterns. Cell Reports. 2016 3; 14(9):2142–53. doi: 10.1016/j.celrep.2016.02.013 26923597 10.1016/j.celrep.2016.02.013

[27] Koleva PT, Kim J-S, Scott JA, Kozyrskyj AL. Microbial programming of health and disease starts during fetal life: intrauterine origin of meconium microbiota. Birth Defects Research Part C: Embryo Today: Reviews. 2015 12;105(4):265–77. 10.1002/bdrc.21117 26663884

[28] Perez-Muñoz ME, Arrieta M-C, Ramer-Tait AE, Walter J. A critical assessment of the “sterile womb” and “in utero colonization” hypotheses: implications for research on the pioneer infant microbiome. Microbiome. 2017;5(1). http://dx.doi.org/10.1186%2Fs40168-017-0268-4 10.1186/s40168-017-0268-4 PMC5410102 28454555

[29] Tissier H. Recherches sur la flore intestinale des nourrissons (état normal et pathologique) Paris: G. Carre and C. Naud; 1900.

[30] Han YW, Ikegami A, Bissada NF, Herbst M, Redline RW, Ashmead GG. Transmission of an uncultivated Bergeyella strain from the oral cavity to amniotic fluid in a case of preterm birth. J Clin Microbiol. 2006 4; 44(4):1475–83. doi: 10.1128/JCM.44.4.1475-1483.2006 16597879 10.1128/JCM.44.4.1475-1483.2006 PMC1448680

[31] Collado MC, Rautava S, Aakko J, Isolauri E, Salminen S. Human gut colonisation may be initiated in utero by distinct microbial communities in the placenta and amniotic fluid. Scientific Reports. 2016 3 22; 6:23129 doi: 10.1038/srep23129 27001291 10.1038/srep23129 PMC4802384

[32] Jiménez E, Fernández L, Marín ML, Martín R, Odriozola JM, Nueno-Palop C, et al Isolation of commensal bacteria from umbilical cord blood of healthy neonates born by cesarean section. Curr Microbiol. 2005 10; 51(4):270–4. doi: 10.1007/s00284-005-0020-3 16187156 10.1007/s00284-005-0020-3

[33] Aagaard K, Ma J, Antony KM, Ganu R, Petrosino J, Versalovic J. The Placenta Harbors a Unique Microbiome. Science Translational Medicine. 2014 5 21; 6(237):237ra65–237ra65. doi: 10.1126/scitranslmed.3008599 24848255 10.1126/scitranslmed.3008599 PMC4929217

[34] Jiménez E, Marín ML, Martín R, Odriozola JM, Olivares M, Xaus J, et al Is meconium from healthy newborns actually sterile? Research in Microbiology. 2008 4; 159(3):187–93. doi: 10.1016/j.resmic.2007.12.007 18281199 10.1016/j.resmic.2007.12.007

[35] Breitbart M, Haynes M, Kelley S, Angly F, Edwards RA, Felts B, et al Viral diversity and dynamics in an infant gut. Research in microbiology. 2008;159(5):367–73. doi: 10.1016/j.resmic.2008.04.006 18541415 10.1016/j.resmic.2008.04.006

[36] Anderson EJ. Prevention and treatment of viral diarrhea in pediatrics. Expert review of anti-infective therapy. 2010;8(2):205–17. doi: 10.1586/eri.10.1 20109050 10.1586/eri.10.1

[37] Ramani S, Kang G. Viruses causing childhood diarrhoea in the developing world. Current opinion in infectious diseases. 2009;22(5):477–82. doi: 10.1097/QCO.0b013e328330662f 19633550 10.1097/QCO.0b013e328330662f

[38] Tate JE, Burton AH, Boschi-Pinto C, Steele AD, Duque J, Parashar UD, et al Estimate of worldwide rotavirus-associated mortality in children younger than 5 years before the introduction of universal rotavirus vaccination programmes: a systematic review and meta-analysis. The Lancet Infectious diseases. 2012;12(2):136–41. doi: 10.1016/S1473-3099(11)70253-5 22030330 10.1016/S1473-3099(11)70253-5

[39] World Health Organization-W. Diarrhoeal disease. 2013. p. Fact sheet N°330.

[40] Milani C, Hevia A, Foroni E, Duranti S, Turroni F, Lugli GA, et al Assessing the Fecal Microbiota: An Optimized Ion Torrent 16S rRNA Gene-Based Analysis Protocol. Quince C, editor. PLoS ONE. 2013 7 15;8(7):e68739 doi: 10.1371/journal.pone.0068739 23869230 10.1371/journal.pone.0068739 PMC3711900

[41] Salter SJ, Cox MJ, Turek EM, Calus ST, Cookson WO, Moffatt MF, et al Reagent and laboratory contamination can critically impact sequence-based microbiome analyses. BMC Biology. 2014;12:87 doi: 10.1186/s12915-014-0087-z 25387460 10.1186/s12915-014-0087-z PMC4228153

[42] Schloss PD, Westcott SL, Ryabin T, Hall JR, Hartmann M, Hollister EB, et al Introducing mothur: Open-Source, Platform-Independent, Community-Supported Software for Describing and Comparing Microbial Communities. Applied and Environmental Microbiology. 2009 12 1;75(23):7537–41. doi: 10.1128/AEM.01541-09 19801464 10.1128/AEM.01541-09 PMC2786419

[43] Schloss PD, Gevers D, Westcott SL. Reducing the effects of PCR amplification and sequencing artifacts on 16S rRNA-based studies. PLoS ONE. 2011;6(12):e27310 doi: 10.1371/journal.pone.0027310 22194782 10.1371/journal.pone.0027310 PMC3237409

[44] Quast C, Pruesse E, Yilmaz P, Gerken J, Schweer T, Yarza P, et al The SILVA ribosomal RNA gene database project: improved data processing and web-based tools. Nucleic Acids Res. 2013 1;41(Database issue):D590–6. doi: 10.1093/nar/gks1219 23193283 10.1093/nar/gks1219 PMC3531112

[45] Edgar RC, Haas BJ, Clemente JC, Quince C, Knight R. UCHIME improves sensitivity and speed of chimera detection. Bioinformatics. 2011 8 15;27(16):2194–200. doi: 10.1093/bioinformatics/btr381 21700674 10.1093/bioinformatics/btr381 PMC3150044

[46] Wang Q, Garrity GM, Tiedje JM, Cole JR. Naive Bayesian classifier for rapid assignment of rRNA sequences into the new bacterial taxonomy. Appl Environ Microbiol. 2007 8;73(16):5261–7. doi: 10.1128/AEM.00062-07 17586664 10.1128/AEM.00062-07 PMC1950982

[47] Benjamini Y, Hochberg Y. Controlling the False Discovery Rate: A Practical and Powerful Approach to Multiple Testing. Journal of the Royal Statistical Society Series B (Methodological). 1995;57(1):289–300.

[48] R Core Team. R: A Language and Environment for Statistical Computing [Internet]. Vienna, Austria: R Foundation for Statistical Computing; 2014 http://www.R-project.org/

[49] Bates D, Maechler M, Bolker B, Walker S. Fitting Linear Mixed-Effects Models Using lme4. Journal of Statistical Software. 2015;67(1).

[50] Heim A, Ebnet C, Harste G, Pring-Akerblom P. Rapid and quantitative detection of human adenovirus DNA by real-time PCR. Journal of medical virology. 2003;70(2):228–39. doi: 10.1002/jmv.10382 12696109 10.1002/jmv.10382

[51] Hoehne M, Schreier E. Detection of Norovirus genogroup I and II by multiplex real-time RT- PCR using a 3'-minor groove binder-DNA probe. BMC infectious diseases. 2006;6:69 doi: 10.1186/1471-2334-6-69 16606447 10.1186/1471-2334-6-69 PMC1524786

[52] Logan C, O'Leary JJ, O'Sullivan N. Real-time reverse transcription PCR detection of norovirus, sapovirus and astrovirus as causative agents of acute viral gastroenteritis. Journal of virological methods. 2007;146(1–2):36–44. doi: 10.1016/j.jviromet.2007.05.031 17644197 10.1016/j.jviromet.2007.05.031

[53] Logan C, O'Leary JJ, O'Sullivan N. Real-time reverse transcription-PCR for detection of rotavirus and adenovirus as causative agents of acute viral gastroenteritis in children. Journal of clinical microbiology. 2006;44(9):3189–95. doi: 10.1128/JCM.00915-06 16954246 10.1128/JCM.00915-06 PMC1594742

[54] Lekana-Douki SE, Kombila-Koumavor C, Nkoghe D, Drosten C, Drexler JF, Leroy EM. Molecular epidemiology of enteric viruses and genotyping of rotavirus A, adenovirus and astrovirus among children under 5 years old in Gabon. International journal of infectious diseases: IJID: official publication of the International Society for Infectious Diseases. 2015;34:90–5. doi: 10.1016/j.ijid.2015.03.009 25796432 10.1016/j.ijid.2015.03.009

[55] Allard A, Albinsson B, Wadell G. Rapid typing of human adenoviruses by a general PCR combined with restriction endonuclease analysis. Journal of clinical microbiology. 2001;39(2):498–505. doi: 10.1128/JCM.39.2.498-505.2001 11158096 10.1128/JCM.39.2.498-505.2001 PMC87765

[56] Chu DK, Poon LL, Guan Y, Peiris JS. Novel astroviruses in insectivorous bats. Journal of virology. 2008;82(18):9107–14. doi: 10.1128/JVI.00857-08 18550669 10.1128/JVI.00857-08 PMC2546893

[57] Oberste MS, Maher K, Kilpatrick DR, Pallansch MA. Molecular evolution of the human enteroviruses: correlation of serotype with VP1 sequence and application to picornavirus classification. Journal of virology. 1999;73(3):1941–8. 9971773 10.1128/jvi.73.3.1941-1948.1999 PMC104435

[58] Nix WA, Oberste MS, Pallansch MA. Sensitive, seminested PCR amplification of VP1 sequences for direct identification of all enterovirus serotypes from original clinical specimens. Journal of clinical microbiology. 2006;44(8):2698–704. doi: 10.1128/JCM.00542-06 16891480 10.1128/JCM.00542-06 PMC1594621

[59] Nasri D, Bouslama L, Omar S, Saoudin H, Bourlet T, Aouni M, et al Typing of human enterovirus by partial sequencing of VP2. Journal of clinical microbiology. 2007;45(8):2370–9. doi: 10.1128/JCM.00093-07 17537940 10.1128/JCM.00093-07 PMC1951248

[60] Hu J, Nomura Y, Bashir A, Fernandez-Hernandez H, Itzkowitz S, Pei Z, et al Diversified microbiota of meconium is affected by maternal diabetes status. PLoS ONE. 2013;8(11):e78257 doi: 10.1371/journal.pone.0078257 24223144 10.1371/journal.pone.0078257 PMC3819383

[61] Ardissone AN, de la Cruz DM, Davis-Richardson AG, Rechcigl KT, Li N, Drew JC, et al Meconium Microbiome Analysis Identifies Bacteria Correlated with Premature Birth. PLoS ONE. 2014 3 10;9(3):e90784 doi: 10.1371/journal.pone.0090784 24614698 10.1371/journal.pone.0090784 PMC3948723

[62] Nagpal R, Tsuji H, Takahashi T, Kawashima K, Nagata S, Nomoto K, et al Sensitive Quantitative Analysis of the Meconium Bacterial Microbiota in Healthy Term Infants Born Vaginally or by Cesarean Section. Frontiers in Microbiology. 2016 12 15 10.3389/fmicb.2016.01997 PMC5156933 28018325

[63] Gosalbes MJ, Llop S, Vallès Y, Moya A, Ballester F, Francino MP. Meconium microbiota types dominated by lactic acid or enteric bacteria are differentially associated with maternal eczema and respiratory problems in infants. Clinical & Experimental Allergy. 2013 2;43(2):198–211. 23331561 10.1111/cea.12063

[64] Janda JM, Abbott SL. The Genera Klebsiella and Raoultella In: The Enterobacteria. 2nd ed. Washington, USA: ASM Press; 2006 p. 115–29.

[65] Marcobal A, Barboza M, Sonnenburg ED, Pudlo N, Martens EC, Desai P, et al Bacteroides in the infant gut consume milk oligosaccharides via mucus-utilization pathways. Cell Host Microbe. 2011 11 17;10(5):507–14. doi: 10.1016/j.chom.2011.10.007 22036470 10.1016/j.chom.2011.10.007 PMC3227561

[66] Azad MB, Konya T, Maughan H, Guttman DS, Field CJ, Chari RS, et al Gut microbiota of healthy Canadian infants: profiles by mode of delivery and infant diet at 4 months. CMAJ. 2013 3 19;185(5):385–94. doi: 10.1503/cmaj.121189 23401405 10.1503/cmaj.121189 PMC3602254

[67] Penders J, Thijs C, Vink C, Stelma FF, Snijders B, Kummeling I, et al Factors influencing the composition of the intestinal microbiota in early infancy. Pediatrics. 2006 8 1;118(2):511–21. doi: 10.1542/peds.2005-2824 16882802 10.1542/peds.2005-2824

[68] Rutayisire E, Huang K, Liu Y, Tao F. The mode of delivery affects the diversity and colonization pattern of the gut microbiota during the first year of infants’ life: a systematic review. BMC Gastroenterology. 2016 12;16(1). 10.1186/s12876-016-0498-0 PMC4967522 27475754

[69] Chu DM, Ma J, Prince AL, Antony KM, Seferovic MD, Aagaard KM. Maturation of the infant microbiome community structure and function across multiple body sites and in relation to mode of delivery. Nature Medicine. 2017 1 23. 10.1038/nm.4272 PMC5345907 28112736

[70] Coppa GV, Bruni S, Morelli L, Soldi S, Gabrielli O. The first prebiotics in humans: human milk oligosaccharides. Journal of clinical gastroenterology. 2004;38:S80–S83. 15220665 10.1097/01.mcg.0000128926.14285.25

[71] Human Microbiome Project Consortium. Structure, function and diversity of the healthy human microbiome. Nature. 2012 6 13;486(7402):207–14. doi: 10.1038/nature11234 22699609 10.1038/nature11234 PMC3564958

[72] Reyes A, Haynes M, Hanson N, Angly FE, Heath AC, Rohwer F, et al Viruses in the faecal microbiota of monozygotic twins and their mothers. Nature. 2010;466(7304):334–8. doi: 10.1038/nature09199 20631792 10.1038/nature09199 PMC2919852

[73] Kapusinszky B, Minor P, Delwart E. Nearly Constant Shedding of Diverse Enteric Viruses by Two Healthy Infants. Journal of clinical microbiology. 2012;50(11):3427–34. doi: 10.1128/JCM.01589-12 22875894 10.1128/JCM.01589-12 PMC3486243

[74] Kanungo S, Kim DR, Haldar B, Snider C, Nalavade U, Kim SA, et al Comparison of IPV to tOPV week 39 boost of primary OPV vaccination in Indian infants: an open labelled randomized controlled trial. Heliyon. 2017;3(1):e00223 doi: 10.1016/j.heliyon.2016.e00223 28194449 10.1016/j.heliyon.2016.e00223 PMC5289926

[75] Mayindou G, Ngokana B, Sidibe A, Moundele V, Koukouikila-Koussounda F, Christevy Vouvoungui J, et al Molecular epidemiology and surveillance of circulating rotavirus and adenovirus in Congolese children with gastroenteritis. Journal of medical virology. 2016;88(4):596–605. doi: 10.1002/jmv.24382 26378607 10.1002/jmv.24382

[76] Richardson S, Grimwood K, Gorrell R, Palombo E, Barnes G, Bishop R. Extended excretion of rotavirus after severe diarrhoea in young children. Lancet. 1998;351(9119):1844–8. doi: 10.1016/S0140-6736(97)11257-0 9652668 10.1016/S0140-6736(97)11257-0

[77] Wilde J, Yolken R, Willoughby R, Eiden J. Improved detection of rotavirus shedding by polymerase chain reaction. Lancet. 1991;337(8737):323–6. 1703618 10.1016/0140-6736(91)90945-l

[78] Dey RS, Ghosh S, Chawla-Sarkar M, Panchalingam S, Nataro JP, Sur D, et al Circulation of a novel pattern of infections by enteric adenovirus serotype 41 among children below 5 years of age in Kolkata, India. Journal of clinical microbiology. 2011;49(2):500–5. doi: 10.1128/JCM.01834-10 21123530 10.1128/JCM.01834-10 PMC3043524

[79] Filho EP, da Costa Faria NR, Fialho AM, de Assis RS, Almeida MM, Rocha M, et al Adenoviruses associated with acute gastroenteritis in hospitalized and community children up to 5 years old in Rio de Janeiro and Salvador, Brazil. Journal of medical microbiology. 2007;56(Pt 3):313–9. doi: 10.1099/jmm.0.46685-0 17314359 10.1099/jmm.0.46685-0

[80] Shinozaki T, Araki K, Fujita Y, Kobayashi M, Tajima T, Abe T. Epidemiology of enteric adenoviruses 40 and 41 in acute gastroenteritis in infants and young children in the Tokyo area. Scandinavian journal of infectious diseases. 1991;23(5):543–7. 1662830 10.3109/00365549109105175

[81] Wold WSM, Horwitz MS. Adenoviruses. Knipe DM, Howley PM, editors. Philadelphia2007.

