# Supplementary figures and images for: Evolution in fecal bacterial/viral composition in infants of two central African countries (Gabon and Republic of the Congo) during their first month of life
Source: PLoS One. 2017 Oct 2;12(10):e0185569. doi: 10.1371/journal.pone.0185569 (PMC5624699; doi:10.1371/journal.pone.0185569)

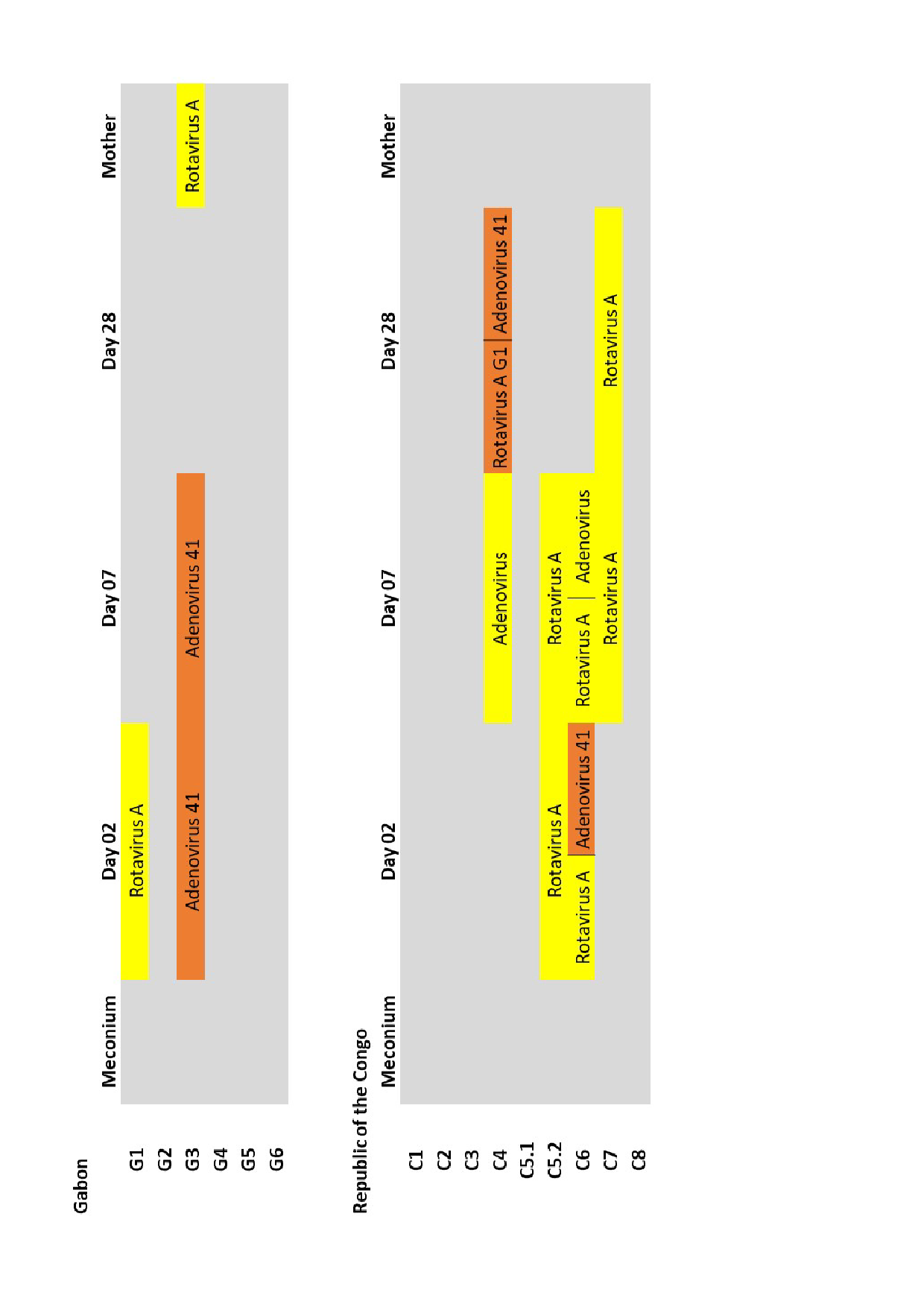

Supplement: S1 Table — PCR and RT-PCR results in fecal samples of 15 infants and their mothers from Gabon and Republic of the Congo. Yellow: positive RT-PCR results, Brown: positives RT-PCR and PCR results. Gray: negative results. (TIF) [file pone.0185569.s001.tif]

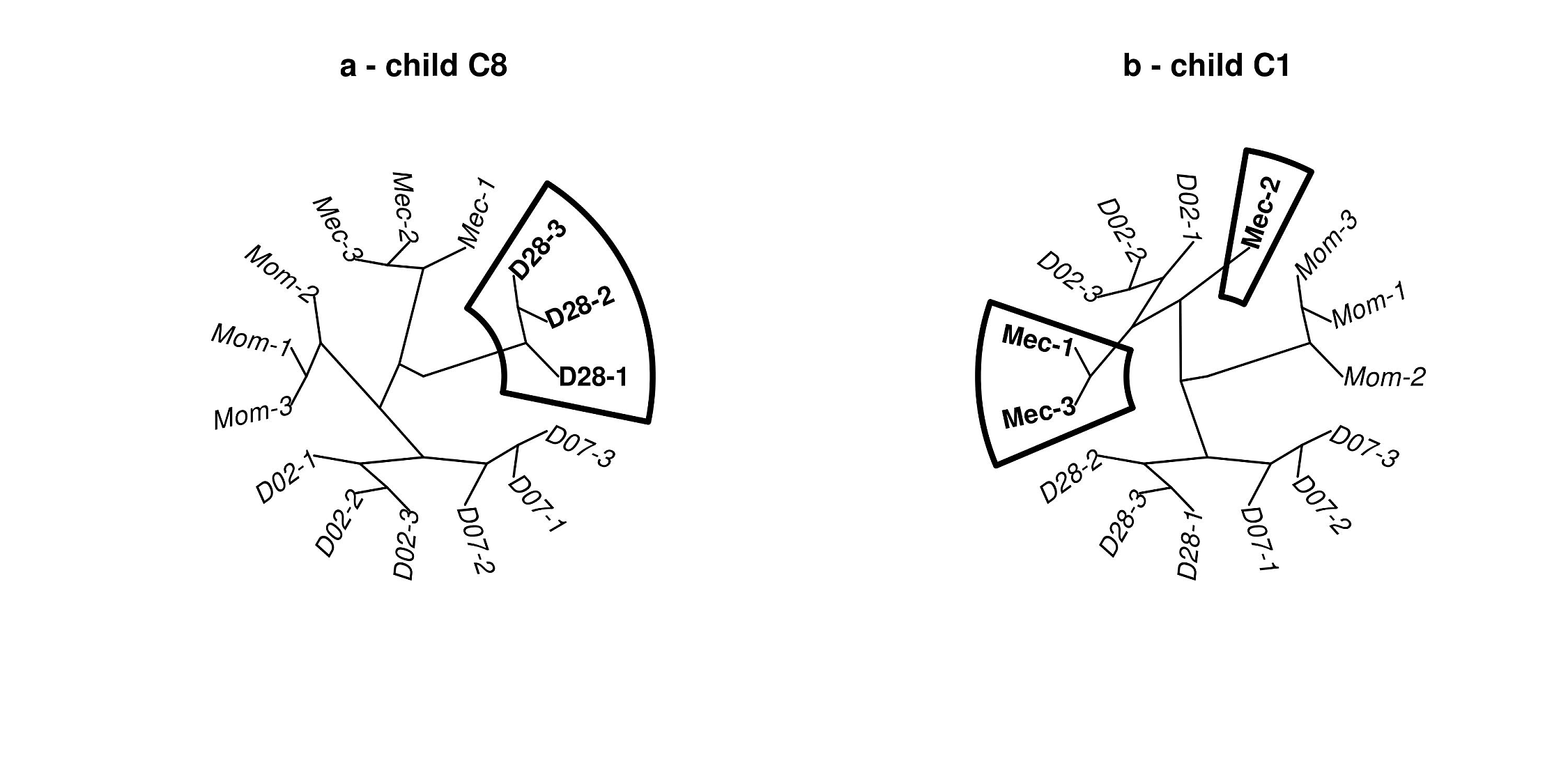

Supplement: S1 Fig — a) Clustering of the genetic samples of child C8 (meconium, D02, Do7 and D28). At all sampling time points, all three genetic samples corresponding to the same fecal sample were in the same cluster. b) Clustering of the genetic samples of child C1 (meconium, D02, Do7 and D28). One of the three meconium genetic samples did not cluster with the other two meconium genetic samples. This genetic sample was discarded. Mec = meconium; Mom = mother; D02, D07, D28 = day 2, 7, 28; 1, 2, 3 = same genetic sample in triplicate. (TIF) [file pone.0185569.s002.tif]

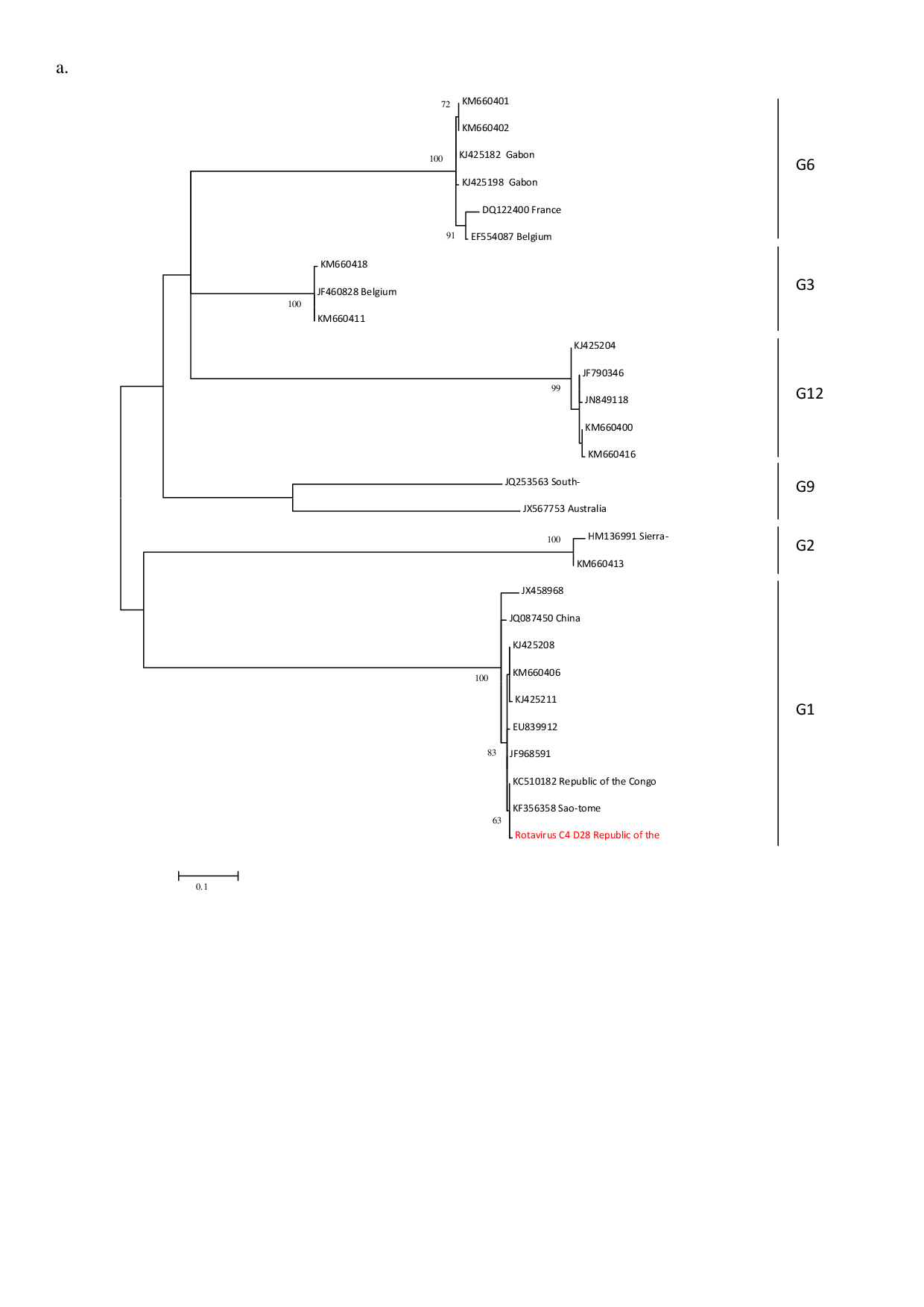

Supplement: S2 Fig — Rotavirus (a) and adenovirus (b) phylogenetic trees. Only bootstrap values ≥60 are shown (500 replicates). Sequences from this work are shown in red and reference sequences are described by their accession numbers and country of origin. (TIF) [file pone.0185569.s003.tif]
